# The associations of anger and hope with project retention decisions: A case study

**DOI:** 10.1371/journal.pone.0283322

**Published:** 2023-04-19

**Authors:** Heba Balatia, Joanna Wincenciak, Trevor Buck

**Affiliations:** 1 The Business School, Edinburgh Napier University, Edinburgh, United Kingdom; 2 School of Education, University of Glasgow, Glasgow, United Kingdom; 3 Adam Smith Business School, University of Glasgow, Glasgow, United Kingdom; Shandong University of Science and Technology, CHINA

## Abstract

The role of emotions and cognition in entrepreneurship and strategic decision-making research has thus far been relatively neglected. In this research, we investigate how anger and hope may influence managers’ project retention decisions. While case studies can never test theories, our research aims to expose the Appraisal Tendency Framework (ATF) to empirical reality in a new context. A Palestinian research context characterized by extreme uncertainty is chosen as one that arguably amplifies the effects of high levels of emotion. Three businesses within a holding company were identified and twelve semi-structured interviews were conducted with managers responsible for strategic decision-making, with data analysed using Content and Thematic Analyses. The emotions of hope and anger were each independently found to be associated with project retention decisions. However, when hope and anger were experienced together, hope complemented a positive association between anger and retention. The AFT proposes that emotions with different valence (i.e., negative anger and positive hope) may be associated with corresponding thought processes (heuristic or systematic) and still result in similar behavioural outcomes. The findings also highlight implications of decision-making under uncertainty, for practitioners who may benefit from differentiating between the positive and negative influences of anger on decisions.

## Introduction

Although initially, the role of emotions did not feature significantly in entrepreneurial research [1], more recent studies have begun to emphasise the influence of emotions on decision-making [2]. Emotions are now as crucial elements of decision-making [3, 4], and, in particular, a possible critical antecedent of project retentions [3].

Few theories have systematically addressed the influences of emotions on judgment and decision-making [5, 6]. The Appraisal Tendency Framework (ATF) [7] is among the first theoretical frameworks that links specific emotions, such as hope and anger, to cognitive appraisals [8], which are proposed to influence different judgmental outcomes [7]. By exposing the ATF to a new empirical context, this study examines the extent to which experiencing anger and hope, in a highly uncertain context, may be associated with retention decisions, where hope may interact with anger.

This study adopts a qualitative case study research method. Twelve semi-structured interviews represent the main source of primary data, intended to investigate the role of anger and hope on project retention decisions. Palestine, which has been facing continuously turbulent political uncertainty [51], is chosen to be the setting of our study. Palestine is a rich setting for studying emotion and project retention decisions in a business context, where the political situation in Palestine may stir up extreme emotions due to high levels of uncertainty, which essentially may affect project retention decisions [35].

Our findings contribute to the literature in four ways. First, studies of emotions in different workplace contexts are under-investigated [9, 10], and our study investigates the severely uncertain context of Palestine, which may be expected to generate heightened anger and hope and thus amplify any associations with project decisions. Second, our empirical findings claim to extend the psychology and decision-making literatures by proposing an association between anger, hope and project retention. This suggests that the ATF may provide a suitable theoretical lens for viewing and explaining retention decisions in a business context. Third, our study investigates possible associations between anger, hope and project retention. There has been no prior empirical evidence of such associations, and hope and anger have not been investigated together. Finally, our results generate propositions for future research.

## Literature

### Emotions in judgements and decisions

Project retention decisions are critical to businesses [11], and traditionally have been addressed mainly from an economic perspective. However, it is now acknowledged that, in addition to financial assessments, decisions may be a function of psychological factors, their resulting emotional costs [12], and the emotional consequences of sense-making and judgement [13]. Emotions may be described as short-term, intense affective states generated by certain triggers, with distinguished cognitive outcomes [14, 15]. Because emotions may be triggered by specific targets or causes [9], they may consequently be connected and interact with behavioural outcomes such as judgments, and decisions [3,8].

Recently, advocates of the ATF have argued that emotions are a personal analysis of meanings and features of an event or a situation relative to personal concerns, needs, goals, and abilities [19, 57]. They have claimed that emotions are associated with a unique set of cognitive and motivational dimensions (appraisals) that link emotions to judgments and decision outcomes [8] and may act as a lens for interpreting subsequent situations [8, 58]. ATF proponents suggest a distinct set of cognitive dimensions differentiates the emotional experience and distinguishes each emotion [8, 16]. This may indicate that emotions of the same valence (i.e. positive or negative) can differently impact cognitive performance [6, 48, 49] Thus, emotions different in valence may also have the same motivational and behavioural outcomes on decision making.

There are six emotion appraisal dimensions that determine the type of emotion: certainty, pleasantness, attentional activity, anticipated effort, control, and responsibility for others [6, 8, 57]. Differences in appraisal tendencies may be relevant to risk perception and attribution [8]. Anger, for example, may be associated with certainty, high control, unpleasantness, and the responsibility for others. Fear, on the other hand, may be associated with low certainty, low control and unpleasantness [6].

The certainty-uncertainty dimension is one significant appraisal dimension. Emotions such as happiness and anger may be linked to certainty in subsequent judgments, which may elicit a better ability to perceive current conditions, control future outcomes and predict future events [17]. Other emotions such as fear, hope, surprise, and worry may be linked to uncertainty and may modify expected returns [17], which may lead to an inability to observe the situation and foresee future occurrences [16, 18].

The appraisal of certainty, proposed by the ATF, is assumed to influence the content and depth of cognitive information search and processing functions [4, 9]. This explains how individuals who experience uncertainty, which is associated with emotions like fear, worry, surprise, and hope, can feel threatened, generate a motive to reduce uncertainty [17], which may encourage the processing of information in a deeper and more systematic way, triggering higher levels of attention [17,16]. In contrast, experiencing certainty associated with emotions like disgust, contentment, and anger, may activate comparatively shallow thought and heuristic processing [16]and be induced by the state of confidence characteristic of these emotions that encourages the use of heuristics [17].

Older theories, such as the affect-as-information and the emotional valence approaches have also explained information processing. The two approaches stipulate that the valence of emotions (i.e., positive or negative) determines the outcome of information processing [18]. This indicates that all negative emotions foster logical and systematic thinking, and all positive emotions foster heuristic thinking [8, 18]. Beyond these two theories and in opposition to them, the ATF finds that emotions of the same valence may impact information processing differently. Thus, emotions of the same valence can differently impact cognitive performance and consequently decision-making outcomes [6, 48, 49]. For example, fear and hope, despite being different in valence, promote systematic processing whereas anger and happiness encourage heuristic processing [7, 16, 24]. It also suggests that old theories of emotions may fail to differentiate the rare judgmental and behavioural outcomes of anger and hope that this study aims to investigate.

Our study selected these two emotions for both theoretical and empirical reasons. Theoretically and congruent with the ATF, anger, despite being a negative emotion, is distinct from other negative emotions since it may activate heuristic information processing rather than systematic thinking. Hope also may promote systematic processing, unlike other positive emotions such as happiness that encourage heuristic processing [7, 16, 24]. These two emotions of hope and anger are very unique despite being different in valence, and appraisal (certainty and uncertainty). This distinguishes this study as it investigates a unique phenomenon. Few academic research has investigated hope in managerial and business contexts [3]. According to Huang et al. (2019) [3] and Snyder’s (2002) [30], hope, is experienced under negative situations and external uncertainty that is compatible with the political extreme uncertainty of our context (i.e. Palestine). Anger, on the other side, is the most often cited and frequently experienced negative emotion [59]. It is selected due to its pejorative use in different management domains [60]. We intend to re-examine the extent to which experiencing anger in an uncertain context may support the advantageous outcomes of anger, such as overriding obstacles [20] and coping with challenging circumstances imposed by a turbulent external environment [29], considering that few recent studies have already reported that anger may have a positive influence on project retention in some contexts, e.g. Geddes et al., 2020 [19]. In addition, the negative experience index predicted that more than four in ten Palestinians were experiencing anger [61]. Finally, there has been no prior investigation of hope and anger together in previous studies.

Empirically, our data shows that anger is found to be a predominant emotion. Notably, hope is also inferred as the second-ranked emotion after anger, given that other emotions emerged from data but are still insignificant due to their low frequencies. This may support Podoynitsyna et al, 2012 [14], where some emotions may be more dominant than others.

Through the lens of ATF, this study examines the possible influence of anger and hope on retention decisions under conditions of severe uncertainty.

### Anger and project retention

Anger “*involves an appraisal of responsibility for wrongdoing by another person or entity and often includes the goal of correcting the perceived wrong*” [19], (p. 29). It is believed to be a particularly influential emotion because it has unique attention-focusing properties [20] and a “*motivational force for justice*” [21], (p.58), which undermines the reputation of anger as an offensive and hostile emotion that lacks reward, attached to negative consequences [22].

Through the lens of the ATF, we propose that anger may be associated with perceived certainty, high personal control, unpleasantness and the perceived responsibility of others for anger [6]. It activates heuristic information processing [16, 23] and is distinguished from other negative emotions, which usually activate systematic decision processing in their patterns, cognitive and action appraisals [24]. Similar to positive emotions, anger may promote optimistic decisions and judgments about future events due to promoting certainty and control of the future [6, 25]. In terms of certainty appraisals, the emotion of anger may be associated with certainty in subsequent judgments due to feeling certain about a situation, a better ability to perceive current conditions and predict future events [6]. Indeed, anger may encourage project retention [26, 27] as it can be useful for survival in certain circumstances [9]. Anger may also enable a better formation and implementation of action plans [27]. This indicates that anger may be connected with the mode of approach and confrontation [20], producing a tendency to override obstacles and restrictions, encouraging change [20, 28], coping with challenging circumstances and taking strong decisions and high risks [29].

### Hope and project decisions

Hope is identified as a positive motivational affective state that is derived from the sense of expecting positive goal energy and deliberate planning to meet goals [30], aiming to improve unfavorable situations in the future [3, 31] and interpreting negative feedback more positively [3, 30].

Borrowing from the ATF, we propose that hope may be associated with uncertain appraisals which may foster uncertainty of judgement, other uncertainty-related feelings, and an inability to observe the situation and foresee future occurrences [6, 18]. These appraisals may activate deeper thought and systematic information processing [16, 17]. Hope may also be characterized by a lack of personal control, which may exist when the individual has little control over situations, where optimistic thinking towards future events is induced [3].

Like anger, hope may also be seen to be associated with project retention [3], where the motivational outcome of hope elicits the anticipation of goal achievement [3, 30, 32], problem-solving, and the exploration of novel situations [33]. This suggests that hope may promote internal motivation to realize goals and the anticipation of working harder to accomplish improved performance [3].

### Hope, anger and project decisions

The two emotions of hope and anger, which have been examined separately in prior research, are traditionally opposed in valence (i.e., hope positive, anger negative). Our study, however, embraces their possible similarity in terms of being stimulated by views of the future, despite their different characteristics in terms of information processing, i.e., hope may foster systematic processing while anger promotes heuristic information processing [18]. Borrowing from Tsai and Young (2010) [25], Lerner and Tiedens (2006) [24] and Lerner and Keltner (2001) [7], we embrace the possibility that anger may induce optimistic thinking towards future events, as well as pessimism. As with the motivational outcomes of anger in future-oriented situations, hope may also motivate persistence, approaching and anticipating goal achievement [3, 30, 32].

## Methods

### Research settings

This research aims to identify the association of anger and hope with project retention through case studies of three projects belonging to an investment holding company operating in Palestine. Palestine is an extremely hostile business context and the world’s most restrictive business environment [34]. It is chosen here to serve as a unique and a rich laboratory where a high level of political uncertainty challenges the survival of businesses, generates heightened emotions, and facilitates this research [35–37].

The three projects operate under conditions of severe uncertainty in Palestine. These conditions are characterised by: 1) a turbulent business climate, 2) the highest national level of commercial risks due to Political uncertainty [62;63] and 3) an extremely hostile context that is influenced by extreme negative institutional pressures [34]. The Israeli-Palestinian conflict mainly contributes to amplify extreme levels of uncertainty [34, 65] which generates distinctive challenges [35] for decision makers to support continuation in an environment where project failure is common [64, 66]. This may be comparable to other uncertain contexts as Afghanistan and Congo, where uncertainty did not restrict business persons’ desire to continue [36, 67].

Located in the Middle East, Palestine is a geographical area occupied by Israel since 1967. It comprises the West Bank (WB, including East Jerusalem) and the Gaza Strip (GS) [68] that are landlocked regions in arguably the most conflicted area in the world [35]. It has also been regulated by the Palestinian National Authority (PNA) from 1993 according to the Oslo Accords [68]. Oslo accords granted the PNA the right to have a civilian and security control of area (A), a civilian control over area (B) in the west bank, and no civilian and security control over area (C), accounting for 61% of the west bank. The WB and GS have been separated politically and geographically and struggle from Israeli controls and restrictions. GS specifically has been under Israeli siege since 2007, with continuous attacks and wars between 2008–2009 and the split between the Palestinian parties in 2007 [34].

This research investigates a leading Palestinian investment company that is an international publicly-held corporation and a major employer in Palestine [38]. It targets three projects affiliated to the holding company, coded as NP, JC and MASH.

The NP project is a date (fruit) production subsidiary in Jericho City specialising in producing Medjool dates and exports to 22 countries around the world [38]. NP managed to achieve success despite the lack of control over water resources due to the Israeli control over them, in addition to the need for permissions from the Israeli side to drill artesian wells to irrigate farms. The Medjool palm is planted in very few locations around the world with distinctive climate conditions, e.g. on the banks of the Jordan valley in Palestine, Israel and Jordan, in some areas in the Maghreb and some parts of California. Medjool dates are the most expensive in the world, and global demand still exceeds supply despite high prices and Palestinian, Jordanian, Israeli, Moroccan and Californian production.

JC is a 5-star hotel in Bethlehem established in 1914. The hotel is a historic palace, a remarkable Palestinian landmark and the first hotel to be built in Palestine. The hotel was operated through an international operator for fifteen years, within an international franchising hotel group operating in the Middle East. However, the hotel continued under local administration after a hostile attack by Israelis on the hotel, which resulted in the forced expulsion of the international operator from the country. The hotel was closed temporarily four times for many reasons. Firstly, the construction of the segregation wall in Bethlehem near the hotel. Secondly, the hotel is also located between two refugee camps (Aza and Ayda) which are two flashpoints between Palestinians and Israelis, resulting in continuous clashes at the entrance to the hotel. The hotel has survived nevertheless.

MASH is a hotel located in Gaza City. The hotel was operated by two international operators sequentially. However, the first international operator cancelled the contract due to the uncertain political situation before the soft opening of the hotel. The second international operator did not manage to survive for more than a year and a half due to weak demand, a lack of financial resources and the semi-collapsed economy. In addition, the cultural difference resulted in heavy losses and consequently the termination of the contract. A dramatic shift occurred later towards self-management through a local administration. The project passed through three phases of temporary termination and one partial termination due to the continuous Israeli attacks and wars on Gaza between 2008–2009, the split between the Palestinian parties in 2007 and the siege on Gaza in 2006 but then managed to continue.

### Research design

As this research examines emotions in the context of decision-making, qualitative methods are appropriate for a deep understanding of multi-faceted perspectives of individuals collaborating within organizations [39]. To obtain a more contextualized and richer examination [40] of affective decision-making in the unique Palestinian context, we applied a multiple case study research method by conducting semi-structured interviews with twelve managers of three projects/subsidiaries belonging to a single holding company. This choice of multiple case studies reflects theoretical replication and triangulation of informants [41]. This means that this study does not aim to study causes and effects of anger and hope but rather the association of these emotions with decision-making.

### Participants

The twelve participants in the study were decision-makers in the top management of the three projects and of the HQ of the leading Palestinian investment company (see Table 1). To determine our sample, we utilised the purposive sampling technique to recruit and select the informants. In terms of recruitment and casing, the first researcher formally contacted a top-level manager in the holding company via email requesting access, which was granted following a short interview. To conduct the casing process, the first researcher visited the company and accepted some suggestions concerning the choice of case studies and interviewees. The first researcher also reviewed the annual reports of the holding company in order to choose the most suitable projects for the study. Together, the first researcher and the manager avoided choosing projects that the holding company had some shares in but did not manage or contribute to their establishment. The choice of projects was based on targeting projects that were still operating despite high uncertainty and others that continued due to some indicators of psychological reasons based on the narrative of the holding company. The author borrowed from the Fang He et al. (2018) [52] categorisation of business situations, where the projects represent two of its categorisations (i.e. projects that continued due to being successful or underperforming). Indeed, not only did the triangulation of data resources, i.e. through using the annual report of the company, contribute to enriching the understanding of authors about the context and cases, but also supported the casing process by providing information about the background of each project and some indicators about its current performance.

**Table 1:** Interviewees’ profile.

Project Name	Interviewee Job Title	Years of Experience	Interview Date	Interview Time
Top management of the holding company	CFO	27	12th June 2019	45 Minutes
	Chief Development Officer	18	12th June 2019	30 Minutes
	Deputy General Manager	25	12th June 2019	1 Hour
NP	The Former Head of Business Development	7	12th June 2019	47 Minutes
	Former Chairman of NP’s BOD	27	25th May 2019	51 Minutes
	Business Development and Operations Manager at NP	16	23rd May 2019	45 Minutes
MASH	Maintenance Manager	18	23rd April 2019	1 Hour and 10 Minutes
	The Financial and Administrative Manager of MASH	26	24th April 2019	44 Minutes
	Room Division Manager	4	24th April 2019	40 Minutes
JC	General Manager of JC	20	2nd June 2019	30 Minutes
	Public Relations Manager	11	1st June 2019	1 Hour
	Room Division Manager	7	1st June 2019	48 Minutes

Source: Author.

The three projects for the case study were mutually agreed upon between the first researcher and the top management. Next, three respondents from the top and middle management of each project were recruited and three other respondents from the board of directors of the holding company were chosen. This implies that the authors relied on the triangulation of informants, by approaching interviewees from different operational levels within the company (the top and middle management). Data collection was ended when data reached saturation by conducting twelve interviews. These participants were chosen because they represent the top of the hierarchy of the projects, they are well educated, have a long experience with the projects, most of them joined the project from inception and are considered the most knowledgeable of these projects. All interviewees are males and have between 7–27 years of work experience. The interviews were arranged with the support of the holding company administration and all projects but one (MASH, which took place remotely) took place in person, between 23 April and 12 June 2019. Interviews’ durations were between thirty minutes and one hour and ten minutes and no one dropped out from interviews or refused to participate.

### Materials

The first researcher conducted the interviews. Only interviewers participated in the interviews and no one else was present in the place where interviews were conducted. Also, there is no connection or a relationship between the first researcher and the company or the interviewers before the beginning of the study and the data collection stage. The first researcher contacted the company and interviewees officially for the purpose of the research during the preparation for the data collection stage and during conducting the interviews. The interview guide containing the research protocol is available as a S1 File (see Appendix 1 in S1 File). Interview questions are also available in Appendix 2 in S1 File. Before interviews, interviewees were briefed about the nature of the research and provided with an electronic copy of the Participant Information Sheet and the Consent Form (See S2 File). Participants provided written consent to take part in the study, which was later confirmed verbally at the start of the interview. Interviews were recorded using two voice digital recorders and were only accessible by the researchers. The first researcher asked open-ended questions to guide the conduct of each interview in hope to understand hope, anger and retention decisions from interviewees’ narratives, their diverse perceptions, their own interpretations, and their fluent description of their emotions. The first researcher also engaged with interviewees’ narratives to create rapport and showed enthusiasm for understanding the context and for reinforcing accurate and persistent narratives. Given the sensitivity of the topic, probing questions were designed to cautiously capture experiences, feelings, and behaviours. The first researcher deliberately did not mention any explicit emotions, leaving participants to spontaneously clarify their emotional reactions and behaviours.

### Ethical considerations

An ethical approval for this research was obtained from the Ethics Committee for Non-Clinical Research Involving Human Subjects of the College of Social Sciences at the University of Glasgow (application number: 400180139). As part of considering the ethical standards, the researchers granted written and oral consent from the interviewees. Interviewees signed a consent form that grants only the authors to access the data. This means that interviews cannot be published to the public because this may lead to breaching the research ethics policy of the University of Glasgow.

### Data analysis

The interviews were first transcribed in Arabic and then translated into English by the first author. With great caution, the first researcher addressed the translation of transcripts, specifically emotions. To do so, she, firstly, relied on two well-established prototypes of emotions in the psychology literature, e.g., the emotions’ prototype approach [31] and the emotion system [32]. She then adopted “contextualising translation “, where context-specific aspects, cross-border and cross-cultural differences were captured yet the richness of data was not lost [42].

This aimed to avoid the identical translation of transcripts and rather to translate transcripts in a ‘target-culture-adequate way’ [53]. This kind of translation was crucial to the investigation and analysis of emotions, specifically anger within the Palestinian culture. This research believed in the importance of the reflectivity of the researcher, i.e. her translation reflected her identity, personal experiences and norms as being Palestinian and her preferred strategies, understanding of the source and target culture, and perceptions of the specific social situations that shape her views, word choices and finally her findings [42]. This way the first author became an active co-producer of the transcripts as the translation process become the data itself, the source of theoretical insights and a way to understand interviewees [42].

Arabic words for each emotion and their subcategories (i.e. as stated in the emotions’ prototype approach of Shaver et al. (1987) [31]) were compared with English words of emotions in terms of their proximity in valence, characteristics and meaning following the circumplex structure of core affect of Russell (1980) [54]. The basic and subcategories of anger as classified in Shaver et al. (1987) [31] contributed to an accurate one-to-one translation of hope and anger and their subcategories. The translated transcripts were then reviewed by the third author, an independent translator and editor. This intended to ensure an accurate translation of meaning from Arabic to English and conversely, specifically to emotions. The coding process and themes were identified after data collection and relied greatly on what has emerged from the field. However, the coding process and later on findings relied on iteration between the data and the recent literature. Triangulation of investigators was also critical to the coding process of data. Each of the authors coded the interviews individually. The authors verified the coding process and approved the similarity in the coding process. When there was a dispute, the authors discussed it together and agreed on a suitable solution. The data analysis process applied Thematic and Content Analyses to the three cases.

To conduct the Thematic Analysis, we followed Braun and Clarke (2006) [43]. Interviews were transcribed and labelled, themes were categorised, and hierarchies of themes were identified. The coding process occurred at three main levels (i.e., 1st and 2nd order codes, aggregate themes) as follows:

1. Generating initial codes, where the 1^st^ order codes were created to develop patterns in meaning and form the potential themes. For instance, joy, fear, sadness, anger and their subcategories were examples of the 1^st^ order codes. In addition, environmental and situational drivers behind retention decisions were also examples of the 1^st^ order codes.
2. 1^st^ order codes were then developed and reviewed by abstracting the 2^nd^ order codes. For example, the explicit and implicit joy, fear, sadness, anger and their subcategories were split into the 2^nd^ order codes of “*negative and positive emotions*” and the environmental and situational drivers behind retention decisions were developed into the “*disappointed/satisfied with economic* and *psychological expectations and impacts*” 2^nd^ order codes.
3. 2^nd^ order codes were also refined, defined and combined into the two established themes of “*emotions*” and “*expectations of decision makers*”.

**Fig 1:**
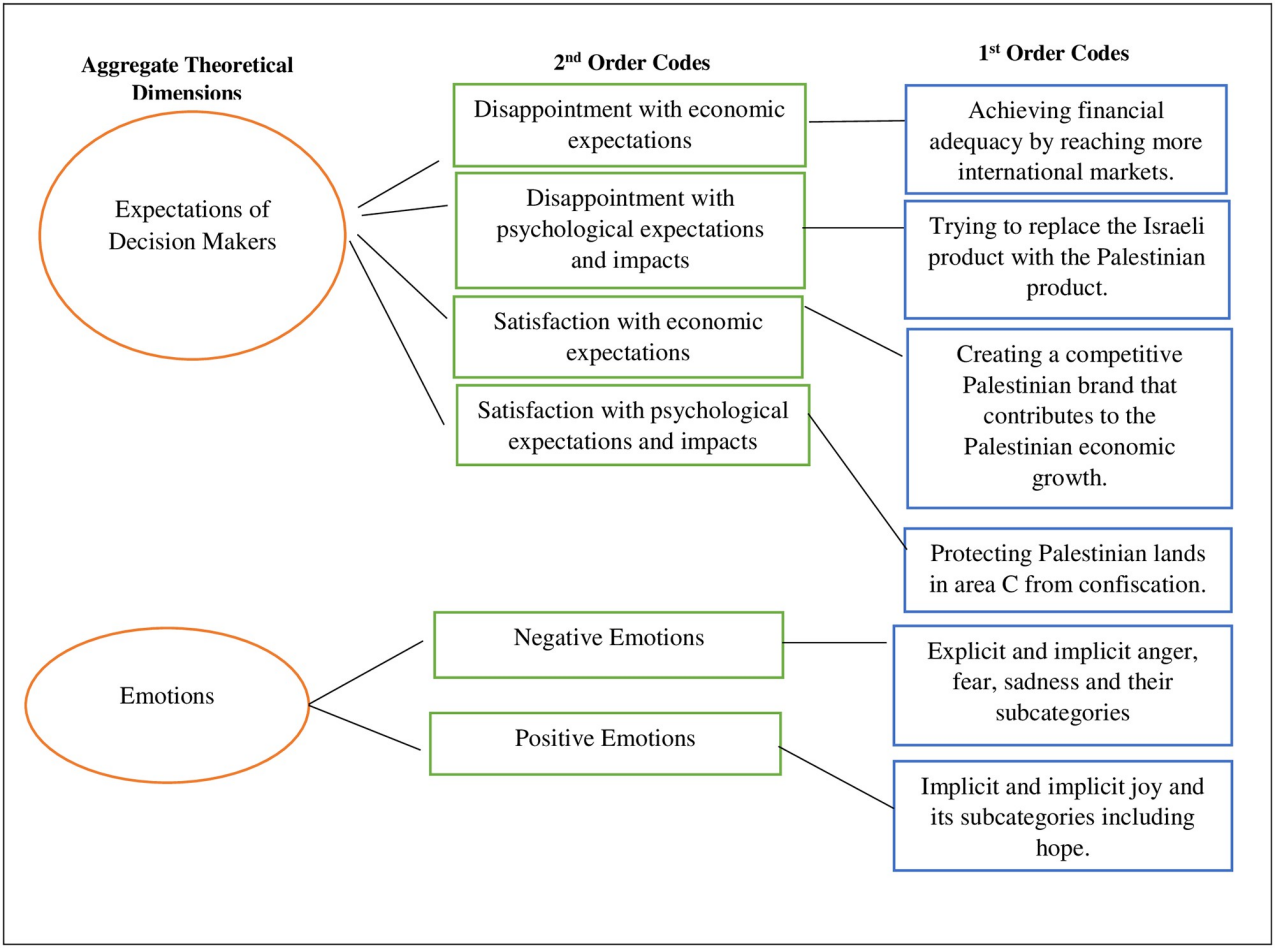
A visualisation of the coding process. Source: Author.

Fig 1 illustrates the hierarchical connections between the aggregate themes of “*the expectations of decision-makers*” and “*emotions*” (which influenced retention decisions) and the first and second codes. “*Disappointed/satisfied with economic* and *psychological expectations and impacts*” were four 2^nd^ order codes for the expectations of decision makers’ theme; and “*negative* and *positive emotions*” were two 2^nd^ order codes for the emotions theme. Finally, *anger* and *hope* appeared as two examples of the 1^st^ order codes for the “*emotions*” theme.

**Fig 2:**
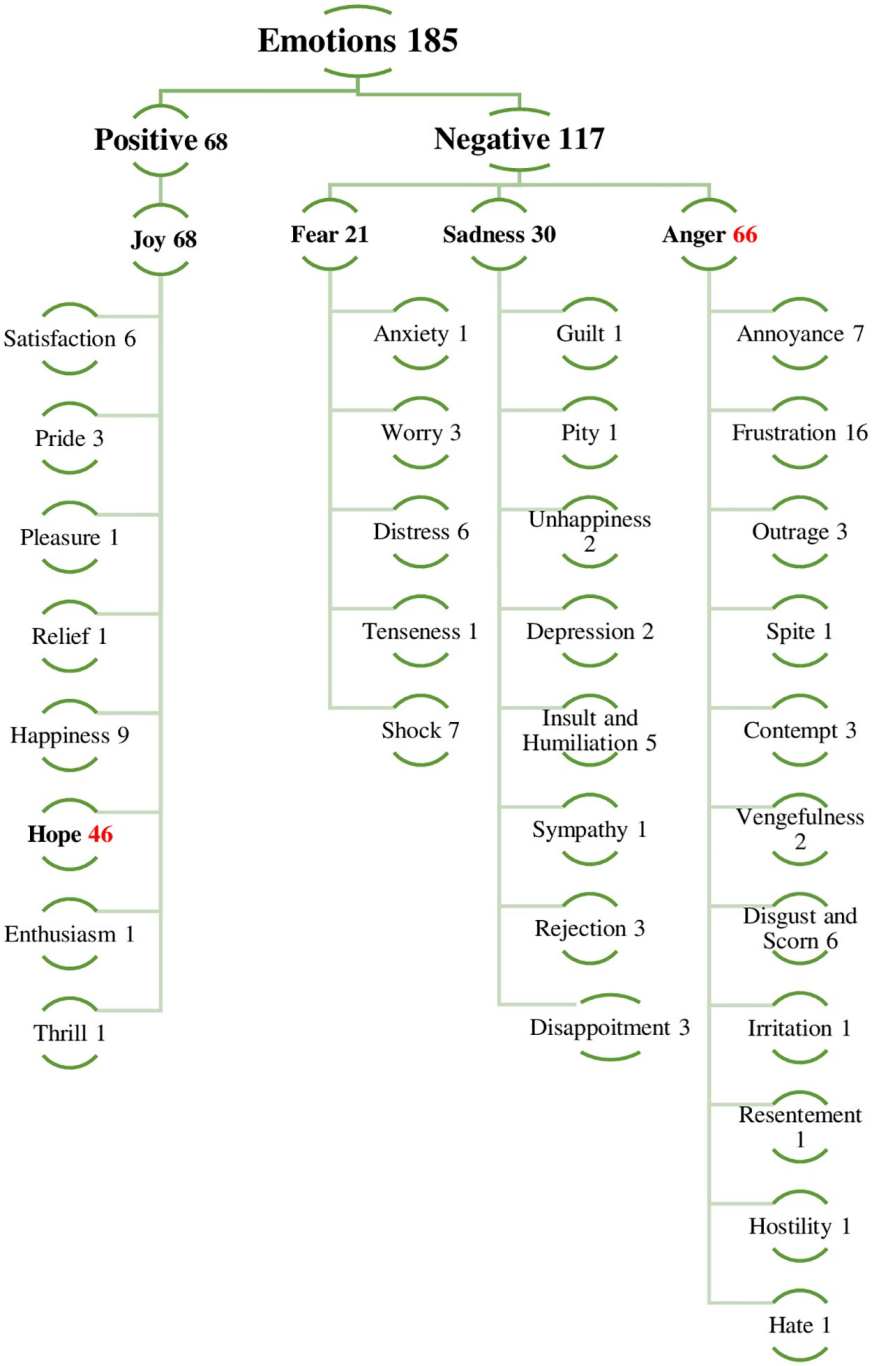
The tree of emotions. Source: Author.

To specifically identify anger and hope in the data, Content Analysis was applied to capturing and analysing the emotions that emerged. We followed several steps. (1) We inductively conducted two rounds of coding to identify emotions. Explicit expressions of emotion were identified in the first round, e.g., “*We were frustrated*” (Frustration—a subcategory of anger), and implicit expressions were identified in the second round; e.g., “*…if the project did not gain profits now*, *there is* ***potential*** *to gain profits in the future…*” (Potential an implicit cue of hope). (2) We calculated the frequencies of the emerged explicit emotions, where anger and hope were cited as the first and second most important emotions respectively. (3) We deductively linked these emotions to their sub-emotions, based on Shaver’s (1987) [31] categorization of emotions. (4) We designed a tree for the emerged emotions (see Fig 2) according to the categorisation of emotions and their subcategories [31], where emotions were classified into negative and positive emotions, then into basic and sub-categories of emotions. For instance, Shaver et al. (1987) [31] stated that *irritation*, *annoyance*, *frustration*, *hostility*, *hate* are subcategories of anger (P. 1067). We support this classification with Greenbaum et al. (2020) [44], where anger and its categories may be interchangeable and equivalent in their emotional responses.

(5) Finally, borrowing from Shaver et al. (1987) [31] and the emotion system of Roseman (2011) [32], anger and hope were analysed according to: a) detailed subjective feelings cues, behavioural and motivational components. b) the triggers (events) and sources (the self, project, the stakeholders, the external environment) of emotions. c) the characteristics of each emotion (explicit vs. implicit expressions of emotions, low vs. high arousal and pleasantness and systematic vs. heuristic thinking). (4) the actions that were taken by managers in order to generate new insights into the role of emotions in project retention decisions (See Table 2).

**Table 2:** The analysis of anger and hope.

Triggering Event (Hassett et al., 2018) or the guiding appraisal (Wang et al.,2018)	Source (Individual, Corporate, Shareholder) (Hassett et al., 2018)	Type of Emotion (Wang et al.,2018)	Subjective Feeling Component (Roseman et al.,2011) (Wang et al.,2018)	Behavioural Component (Roseman et al.,2011) (Wang et al.,2018)	Motivational Component (Roseman et al.,2011) (Wang et al.,2018)	Action	Quote
Unfair treatment of Palestinian labour and the unjust restrictions imposed on NP project.	Individual emotions towards the external business environment.	Anger.Characteristics: Implicit expression of negative emotions. High Arousal, Low Pleasantness (Russell, 1980). Heuristic thinking (Lerner et al., 2015).	The judgment that the situation is illegitimate, wrong, unfair, contrary to what ought to be, violation of expectations, an interruption of goal-directed activity (Shaver et al.,1987). Injustice and explosive (Roseman et al.,2011).	Narrowing attention, to exclude all but the anger situation, suppressing anger and redefining the situation (Shaver et al.,1987).	Moving against others (Shaver et al.,1987).	Project retention.	“Many challenges are directly linked to the Israeli occupation directly as **wells’ licensing, permissions to the plantation, lands confiscation, exporting limitations and challenges that are related to importing some kinds of fertilizers and insecticides**…” NP01
NP project’s endless challenges.	Individual emotions towards the project.	Frustration as a subcategory of anger. (Shaver et al.,1987)Characteristics: Explicit expression of negative emotions. High Arousal, Low Pleasantness (Russell, 1980). Heuristic thinking (Lerner et al., 2015).	Facing obstacles and tension (Roseman et al.,2011), things not working as planned, real psychological pain (Shaver et al.,1987) and feeling explosive (Roseman et al.,2011).	Exertion of effort (Roseman et al.,2011) Redefining the situation and suppressing the anger (Shaver et al.,1987).	To overcome the situation (Move against it) (Roseman et al.,2011).	Project retention.	“In this project, challenges were endless and not easy …I remember the first two years of the project, the mortality rate of saplings was 90% … and this is alone was enough to stop the project and for us to reach **frustration.** We started to think that we are wasting our time, money and effort. But we had this insistence and determination that the project must continue and that we learn from our faults.” NP01
High country, industry and political risks	The external environment and the project.	Potential as implicit cue of hope (Roseman et al.,2011).Characteristics: Implicit expression of positive emotions. Systematic thinking (Lerner et al., 2015).	Potiential and foused (Roseman et al.,2011).	Anticipate and approach (Roseman et al.,2011).	To make something happens and move toward it (Roseman et al.,2011).	Project Retention	*“There is definitely a high risk in this project but also a reasonable return that drives us to invest in such a high-risk product…This means that there is* ***potential*** *in the project*. *Hence*, *if the project did not gain profits now*, *there is* ***potential*** *to gain profits in the future…”* NP02

Source: Author.

Before presenting our results, it is essential to discuss how our research addressed external validity. External validity was addressed in terms of theoretical generalisability, where a case study usually generalises theoretically and not statistically [55, 56]. The inclusion of multiple project cases as a subunit within a single case study allowed an increase in the robustness of findings through the replication across the three projects. Indeed, confirming the results of case studies may be approved only when contextual details are generalizable across multiple cases [70]. This indicates that contextualisation plays a dynamic, multi-layered, and endogenous role in explaining the phenomenon under investigation, instead of being a constraint of this research. A social phenomenon, such as the influence of hope and anger on retention decisions, cannot be explained without understanding its context (Palestine). The contextual approach, which contributes to recognising the distinctiveness and uniqueness of situations and events, states that events (i.e., the uncertain political conditions in Palestine) can change features of contexts and change personalities, and activate certain traits [69].

We confirm in this study that the case study significantly intends to reconcile theory and context and provide a holistic understanding of the determinant factors of the phenomenon. This is basically because minimising contextualisation may result in a misleading or incomplete theoretical explanation, where the context becomes part of the explanation and constitutive of it. Here, we intend to provide situated explanations and not generalisable findings [70].

Context also allows for the transferability of case studies findings to other contexts, where eliminating the possibility of ignoring the outliers, and negative cases may generate important theoretical insights. Generalisability may demonstrate that cases are homogeneous and similar rather than emphasising the heterogeneity across cases [70]. We, therefore, propose that future research could investigate the influence of anger and hope on other under-examined but similar contexts to expand the transferability of our findings. This applies to other recent political turbulence, such as Ukraine, pandemic uncertainties as COVID-19 and economic crises such as the shrinking economies due to COVID-19, while considering the particularity of each context.

This may emphasize that decontextualisation may jeopardise our claimed theoretical contributions through presenting oversimplified explanations, reductionism, and misinterpretation of the phenomenon in terms of social structures, culture, and the uniqueness of the context. This may misinterpret the heterogeneity of cases and promote an identical explanation to all cases, where the phenomenon become detached from time, space, and the settings we study [70].

## Results

The extremely uncertain economic and political situation in Palestine has promoted extreme emotions, and anger and hope were the two most cited emotions that support retention decisions. Applying the claims of Lerner and Tiedens (2006) [24] regarding anger as a response to injustice and violations of rights, we report that such an unstable environment evoked intense feelings of anger in the context of all three projects (NP, MASH and JC). It was also evident that most decision makers expressed hope.

### NP project

The NP project operates in a turbulent agriculture sector and can be described as being hindered by Israeli restrictions, which challenge the continuation of the project and interrupt its operations. Despite this, our findings showed that NP decision-makers tended to favor project retention, experiencing anger and hope.

Anger was provoked by the Israeli agricultural restrictions imposed on Palestinians. They increased decision makers’ desire to persist with the provision of alternative job opportunities for Palestinians, to protect Palestinian lands in Area C from confiscation, and to create a competitive Palestinian brand. This may have been an attempt to consequently suppress the anger and redefine the situation. Indeed, experiencing illegitimate situations and being harmed, from the Palestinian perspective, appeared as implicit cues of anger for NP01 decision maker:

*“…The Israeli control over water resources significantly harmed us and denied us our right to exploit such resources in our agricultural activities…”*

NP02 also reported an implicit cue of anger, where he explained experiencing unexpected events due to the Israeli restrictions on water resources. These plantation conditions contributed to decreased productivity but did not lead to project termination.

*“…The plantation conditions that we provide for our trees are not comparable to what Israelis provide for their farms and trees especially in terms of water resources… This has affected our productivity expectations…”*

Anger was also implicitly generated from feeling unjust and experiencing the interruption of goal-directed activities. Despite this, anger did not weaken their desire to retain the project. NP02 decision maker reported that:

*“… the conditions at plantations are very restricted by Israelis and not comparable to the unrestricted settings and the privileges that Israel provides for their farms especially in terms of water resources”*

Anger was also triggered by political, country and industry risks. However, NP decision makers were satisfied with the fulfilment of their economic expectations and the project achieved some of their financial expectations, despite these risks. On decision maker of NP said:

*“*… *Investing in Palestine is in itself risky and the investment*, *political and country risks are high in comparison with any other country around the world …”*

NP03

NP03 explained that anger was generated by high industry risks, specifically, where agricultural projects were riskier than in other sectors. He experienced frustration, one subcategory of anger. He stated:

*“In this project*, *challenges were endless and severe … the mortality rate of planted saplings was 90%*… *And this alone was enough to stop the project and to cause frustration … we started to think that we were wasting our time*, *money and effort…”*

These risks contributed to delays in some operations and restricted others, e.g., being forbidden to grant permission for the planting and irrigation and using certain types of fertilisers, in addition to the lack of control over water resources. NP01 further described these challenges and further clarified how anger was provoked by the interruption of goals and plans.

*“We do not have any control over water resources*, *e*.*g*. *permission to drill artesian wells to irrigate our farms*. *This obligates us to buy water from other resources such as privately-owned wells*, *which increase the cost of production…”*

High production costs and Israeli competition on external markets were another two challenges. NP decision-makers illustrated this in the following quotations:

*“… We expected a different level of productivity for the palm tree than we have experienced in reality…The nearest market to benchmark is the Israeli market*. *But we discovered that the facilities that are provided for the Israeli farmer are not available to the Palestinian farmer…they compete with us in similar external markets”*

NP02

Although the NP project was harmed by these risks and challenges that were provoked anger, NP decision makers decided to continue. NP03 clarified:

*“The reasons for our continuation are our high belief in this project … We do not wait for revenue in the short-term but consider it a strategic project even if it only starts to gain revenue after 10 years”*

NP03

Despite experiencing anger, decision-makers were equipped with hope, associated with retention. Hope, which was also generated by the future economic potential of the NP project, was essential to support retention by these decision-makers. Some NP decision makers experienced ‘having potential’ as an implicit cue of hope.

*“The continuation decision is due to the high potential of the project’s growth*. *It is considered to be a promising business*, *and this is why I think the decision was to retain the project”*

NP01

Indeed, hope was associated with retention decisions, when NP decision makers were satisfied with the achievement of the economic expectations of their projects. NP02 decision-makers praised the economic feasibility of the project and their ability to exploit unique climate characteristics to achieve the economic advantages of Medjool dates.

*“One advantage of Medjool dates is the need to be grown in specific weather conditions that exist in only two or three areas around the world (i*.*e*., *California*, *the Jordan Valley and the Maghreb)… by planting and producing dates in this area … the company will feasibly start to enjoy a profit starting from 2020…”*

Another decision maker described how the project reached international markets, creating a competitive brand that contributed to Palestinian economic growth.

*“…Creating a Palestinian brand that is capable of domestically competing with Israeli products and also being exportable… to 20 or 25 countries around the world … providing a hard currency with a new source of revenue to the country and contributing to economic growth…”*

NP03

Supported by hope, NP decision-makers also achieved some of the economic expectations of the project. One NP decision maker referred to their high current and future profit margins, given the high global demand for Medjool dates internationally.

*“… we will reach production optimization because our trees are growing*: *there is a demand for our products in the global markets and stable prices”*

NP02

In conclusion, NP decision makers relied on hope, that complements the positive motivational outcomes of anger to promote the retention decision.

### JC project

The JC project is a hotel operating in Bethlehem City. JC decision-makers also experienced anger and hope. Anger and hate, a subcategory of anger, which was associated with the continuation of the project, were experienced by JC03:

*“No one was happy with the closure of the hotel*. *We felt sad and angry although we could do nothing to support the hotel*. *Also*, *we felt that hatred was intensified towards the Israeli occupation due to such challenges that they imposed on us … However*, *these emotions deepened the desire of employees to put in more effort to support the survival of the hotel”*

JC03

Some JC decision makers experienced hostility as an extreme component of anger. They described their anger after Israelis attacked the hotel, which left them with limited control over the business’s operation. However, this still encouraged them to start again, continue and improve:

*“Israelis get used to provoking and attacking us with tear gas and sewage and chemical water*… *Thus*, *when the Israeli soldiers blocked the hotel’s road and attacked the hotel and its customers with tear gas*, *the hotel lost its reputation as a safe place to host people”*

JC02

With four periods of temporary closure due to Israeli assaults on JC, JC decision-makers, over the years failed to satisfy some of their economic expectations caused by the unstable political uncertainty during most of the project life and the lack of demand due to political and economic instability. A senior manager explained:

*“The main reason for project losses in Palestine is the political situation and wars*. *All projects*, *regardless of their type*, *are affected by this factor*. *However*, *some sectors are affected more than others…”*

POR01

JC decision-makers also were dissatisfied with the limited scope of their economic achievements in the local market. JC01 mentioned:

*“The target that we aim to achieve is a worldwide historic hotel*, *which requires competing worldwide rather than locally…For me personally*, *I think that no managers are satisfied with the company that they manage…I might be satisfied to a certain extent*, *but I did not achieve all my targets…”*

JC01

Decision-makers also experienced hope to overcome their anger and to encourage continuation. Hope was described through their ambition to continue, which is also an implicit cue of hope. Their aim to achieve their future financial rewards strongly supported retention.

*“Closing the hotel permanently was never an option but the decision to continue was*, *and still is*, *our ambition as continuation might drive us towards future financial rewards*. *After waiting patiently for a long time*, *we finally started to achieve profits”*

JC03

Despite the years of loss, turbulent political and economic circumstances, and the direct Israeli attack on the hotel, the JC project eventually managed to harvest the fruits of hope and to accomplish its economic expectations, specifically, an improvement in economic performance, profitability and managed to continue after years of anticipation. According to the response of JC01:

*“After 20 years of waiting and expecting*, *we have finally achieved more profit and performance than we expected…we have achieved better results with lower costs than we expected*.”

JC01

Hope motivated the continuation of the JC project. JC decision-makers explained that they achieved important psychological impacts related to supporting the Palestinian economy by creating job opportunities for the local communities of Bethlehem, during the underperforming stage of their project.

*“Another aim is to provide local jobs for the people of Bethlehem*. *The hotel has provided 200 job opportunities to support the lives of 200 families in Bethlehem*… *We have achieved this goal*!”

JC03

In terms of prestige, they also achieved worldwide and regional prizes, taking advantage of the historical and religious location of Bethlehem city, as the home of three religions, which encouraged project continuation. They also achieved the target of protecting the Palestinian heritage and identity:

*“…We also took advantage of identifying Palestine as the home of three religions that pilgrims visit from all around the world*. *So*, *it was very important to have a presence in Jerusalem and Bethlehem”*

POR02

*“We were very happy when the hotel achieved the prize for worldwide Historic Hotel*. *We were happy because our efforts did not go down the drain and our accomplishments are honoured”*

JC03

This demonstrates that the retention decisions of JC decision makers relied primarily on hope and positive outcomes of anger to continue their projects.

Taking these three projects together, this research illustrates how anger and hope may be associated with project commitment and continuation. Hope may have been associated with project commitment by complementing anger in relation to project retention under severe conditions. It also indicates that anger and hope contributed to pushing the firm towards satisfaction with the project, despite many challenging obstacles. In the next section, we build on these findings by discussing relevant prior literature and highlighting our contributions.

### MASH project

MASH decision-makers decided to continue with their project despite barriers, e.g., the Israeli siege of Gaza, wars and the closure of Gaza’s crossing points. MASH decision makers expressed anger and hope.

Annoyance, irritation and frustration (subcategories of anger), were also expressed towards this project. As in the following quotations, the analysis showed a strong psychological attachment, commitment and loyalty to the project, which contributed to promoting a mostly positive role of anger in its association with project retention. One MASH decision maker expressed these emotions:

*“We are not angels or gods; we sometimes feel frustrated*, *especially when days pass without any sales*, *given that we have running costs anyway and no reward at all*. *However*, *we look forward*, *hoping for returns even if they come late”*

MASH02

*“Sometimes we get annoyed*. *For instance*, *when a decision was taken to decrease the working hours of employees and thus salaries*. *However*, *we supressed annoyance as we got used to these shocks”*

MASH01

MASH decision makers were angry and dissatisfied with reference to their economic expectations due mainly to the political uncertainty and associated economic losses, which was provoked by feelings of injustice. This highlighted the importance of achieving psychological expectations by supressing this anger when the project was underperforming economically. One decision maker expressed this in the following quotation:

*“Unfortunately*, *we did not achieve many financial goals*, *due to…the uncertain political and economic situation that has affected the economic expected returns and profits of the company… this means the holding company cannot exploit all of its capabilities and capacities due to the political and economic restrictions…”*

MASH02

Their dissatisfaction with not being able to satisfy their economic performance expectations were represented by their failure to generate a stronger customer base and to achieve economic and strategic alignment and growth in the face of high economic and political uncertainty. Indeed, their anger here was mainly elicited by the interruption of goals. MASH decision-makers described this:

*“The hotel project relied on a feasibility study which assumed that crossing points were open*, *there was an airport in Gaza and that the headquarters of the Palestinian Authority would be in Gaza*. *However*, *we have not achieved any of these initial expectations…”*

MASH01

Anger was also experienced by customers due to the cultural differences and culture shock of international operators’ management. However, the project continued by adopting a localisation strategy for the project. MASH 03 mentioned this in his interview:

*“They did not understand the culture of the country from the client’s side as well as…The general manager annoyed our clients and was annoyed by them for the simplest reasons*! *Studying and understanding clients I guess is extremely important*!”

Anger, expressed by MASH decision makers, contributed to reshaping their decision. They were satisfied with achieving some of their project’s psychological expectations. This was achieved by reinforcing their intentions to invest in Palestine and enhancing its economy by creating job opportunities. We support this with MASH02’s quotation:

*“…We aim to enhance the Palestinian economy in terms of creating job opportunities and entertainment …”* MASH02Driven by positive outcomes for anger, MASH also satisfied some of its psychological expectations. In terms of their achieved performance expectations, decision-makers were proud that they maintained a good customer base and survived, thanks to their strong psychological commitment and loyalty to their project, despite the turbulent political and economic environment. A decision maker illustrated these feelings and opinions:

*“Although our project is still economically under-performing we still believe that continuation is a matter of achieving our financial and non-financial targets…this means we do not work only for the interest of our hotel but also for Palestine*. *We aim to create an iconic company in an underdeveloped and occupied country”*

MASH02

Retention decisions, provoked by anger, was found to be influenced by both personal and project-level commitments. Affective (psychological), normative (moral) and continuous (i.e. no other available job opportunities) commitments. MASH02 described his anger towards the project and the self.

*‘we sometimes feel frustrated especially when days pass without dealing with any customer*…*Sometimes I think of my own interest and I think that I should leave the hotel*, *but I feel that I have a moral commitment towards the company…It is rather a moral*, *ethical and patriotic commitment…”*

MASH decision-makers also expressed hope for a stable political and economic situation, which also supported project retention, since uncertainties were perceived as being temporary. Hope was experienced explicitly by these decision-makers, as illustrated by the following quotation:

*“We look forward*, *hoping for returns even if they come late*. *We usually wait for a glimmer of hope that may come at any time through a political agreement or a new government*, *to revive the market or to gain governmental agencies as our clients…”*

MASH02

Other decision-makers in the top management team also experienced hope. Through project retention, they expected high profits from the MASH project. Hope encouraged them to anticipate future returns for the project, regardless of its current performance. A decision-maker in the top management explained these predictions:

*“… we believe that loss is temporary and we work hard to replace losses with gains and redirect projects into profitability*. *…We hope that the conditions will be more stable in Gaza some time during the coming 24 months*, *hours or less*. *Whenever the conditions become stable in Gaza*, *the movement to and from Gaza will be very active*.*…”*

POR03

This indicates that MASH decision makers relied on hope and the positive anger, to encourage retention decisions.

## Discussion

Our study provides new insights into the association of anger and hope with project retention decisions under extreme uncertainty. In particular, our empirical evidence provides original evidence that turbulent contexts as Palestine may generate extreme emotions, such as anger and hope, which reinforces project retention.

In contrast with some previous research which argues that anger resulting from past events evokes pessimistic consequences (e.g., [22, 45]), our research indicates that anger may prompt optimistic perception regarding future events and circumstances [7, 24]. In line with Huang et al. (2019) [3], Vaestfjaell and Slovic (2013) [46] and Harvey and Victoravich (2009) [47] our findings from the three projects are consistent with the notion that anger may be an encouraging emotion. This means that decision-makers may favor project continuation due to their belief of the possible future benefits of these projects and their ability to mitigate uncertainties, improve project performance and satisfy expectations. These findings are also consistent with Seckler et al. (2017) [26] and Maglio et al. (2014) [27] who identify anger with project retention, and also support Lerner and Tiedens (2006) [24] who consider that the motivational outcomes of anger may be to promote decisive success, confront problematic situations and overcome obstacles.

The influence of anger on project retention is also consistent with the ATF, which suggests that emotions of the same valence can differently impact cognitive performance and consequently decision-making outcomes [6, 48, 49]. Indeed, despite being a negative emotion, anger may be distinct from other negative emotions since it may activate heuristic information processing rather than systematic thinking, which this research links to retention decisions. Based on these arguments, we suggest:

*Proposition 1*: *Anger may be positively associated with project retention*.

In addition to anger, our study has illustrated that the extremely uncertain context of Palestine has generated hope, and thus promoted project retention. This is consistent with Huang et al. (2019) [3] and Snyder (2002) [30] who propose that hope implies that unfavorable situations could be improved in the future and that negative feedback is interpreted more positively. Roseman (2011) [32] also proposes that hope motivates persistence, approaching and anticipating goal achievement.

These findings are consistent with the ATF, whereby hope may promote systematic processing, unlike other positive emotions such as happiness that encourage heuristic processing [7, 16, 18]. Therefore, we suggest:

*Proposition 2*: *Hope may be positively associated with project retention*.

By broadening prior research, our empirical evidence reveals that when anger and hope are experienced together, hope may complement anger and an association with project retention under severe conditions. Within the ATF, hope and anger are recognized as being different in valence (i.e., positive hope, negative anger), but we observe their similarity in terms of their tendency to be associated with project retention despite their different consequences in terms of information processing, i.e. hope fosters systematic processing while anger promotes heuristic information processing [18]. To justify the similar effects of hope with anger, we borrow from Tsai and Young (2010) [25], Lerner and Tiedens (2006) [24] and Lerner and Keltner (2001) [7] who assume that anger also induces optimistic thinking towards future events. As with the motivational implications of anger in future-oriented situations, hope also motivates persistence, approaching and achieving goals [3, 30, 32]. This is consistent with our findings, where both anger and hope promote project retention. Therefore, we suggest:

*Proposition 3*: *Hope may complement anger to reinforce its positive association with project retention*.

## Conclusion

Our research utilises the ATF in an investigation of the roles of anger and hope in decision-making under extreme uncertainty. It also employs a case study that advances the ATF by exposing it to empirical reality in a new context and yields an understanding of the mechanisms by which extreme anger and hope, generated in conflict areas, may affect decision-making outcomes. This means that our findings were consistent with an ATF-based interpretation. Of course, case studies can never confirm theories, but at least ATF was not falsified by empirical observations from a most extreme business environment. Our findings reveal that retention decisions were positively associated with anger and hope. The study concludes that decision-makers may be influenced by anger and hope, to make retention decisions. In this study, we claim four theoretical contributions:

1. Our findings claim to extend the psychology and decision-making literatures by proposing separate influential roles for anger and hope on project retention. The application of psychological theories to a business phenomenon may provide a novel explanation of retention decisions. Indeed, the behavioural and motivational effects of emotions, investigated in the psychology literature and adopted directly from Roseman et al. (2011) [32] and Shaver et al. (1987) [31], may suggest a new theoretical lens through which project retention decisions may be analysed.
2. Our research illustrates the utility of the ATF and claims to extend it towards explaining retention decisions in a fresh business context. While earlier studies adopting the ATF have focused on each specific emotion separately, with limited application to entrepreneurial decision-making, no research has studied the combined association of anger and hope with retention decisions.
3. We also add a fresh context, by assuming that severely uncertain contexts, as in Palestine, may generate raised emotions, including anger and hope, which may promote retention decisions. In doing so, we contribute to an underdeveloped area in the entrepreneurship and decision-making literatures and suggest examining other business phenomena in uncertain and conflicting contexts [50]. This implies that turbulent environments may crucially apply some positive influence on the survival and continuation of corporations in such contexts.
4. Finally, we contribute to the literature by suggesting three propositions for future research. The propositions include a complementary role for hope and anger in favoring retention decisions.

As with any research, our study has some limitations. For instance, gender bias. All interviewees are males as the board of directors and top management of the company under investigation is male-dominated. Also, there may be a methodological limitation. The research relied only on qualitative research methods. Alternative measures of emotions, besides interviews, e.g. surveys, observing facial, vocal expressions and body gestures, may be important and possibly confirm interviewees’ results [57] and enhance their internal validity of the results. Unfortunately, time and access limitations prevent the researchers from involving them in this research.

## Supporting information

S1 File (DOCX)

S2 File Participant information sheet. (DOCX)
