# Supplementary material for: The associations of anger and hope with project retention decisions: A case study
Source: PLoS One. 2023 Apr 19;18(4):e0283322. doi: 10.1371/journal.pone.0283322 (PMC10115263; doi:10.1371/journal.pone.0283322)
Supplement: S1 File — (DOCX) [file pone.0283322.s001.docx]

**Supporting Information1**

**Appendix (1): An Interview Guide**

1. The researcher sent an acknowledgement email to the interviewees, explaining the purpose of the study and the data collection plan. Participants also received an electronic copy of the Plain Language Statement (Participant information sheet) providing details of the study and data use, and the Consent Form. Written consent was obtained prior to scheduling the interview. The details provided to interviewees in the Plain Language Statement are as follows:

‘*This research aims to study the relationship between anger as one specific emotion and the retention/termination decisions. It will also, highlight the influence of sunk cost bias and portfolio considerations on the anger – retention/ termination decision relationship*.’

Please note that other emotions such as hope have emerged from the data. Also, other variables such as the sunk cost effect were investigated in this research but were not considered as part of this manuscript. Also, termination was investigated in this research but it did not appear in the results only temporal termination emerged.

1. Oral consent was reaffirmed at the start of the interview. All the interviews were recorded via two voice digital recorders, and interviewees were informed that these audio recordings were only accessed by the researcher and used to transcribe the interviews.
2. At the beginning of the interview, the researcher introduced herself . Interviewees were informed about the name of the researchers, the job (i.e. researcher), the reasons for doing the research (a PhD student) and the university where the researcher studies (University of Glasgow). She then presented a briefing about the research topic. In this stage and to avoid leading the interviewers towards specific answers, the researcher mentioned that the study is about the influence of emotions and cognitive bias on the retention and termination decisions of managers. She also informed interviewees about what the researcher expected from them during the interview. The researcher tried to create rapport to reduce any tension and create a climate of trust with the interviewees. Then, the researcher asked participants to introduce themselves, confirming their job titles, education and experiences.
3. The researcher asked broad, open-ended interview questions to encourage the interviewees to express their experiences and opinions (see Appendix 2). She tried to give a big space and time for interviewees to narrate their own stories, experiences and emotions with minimal interruption. If the researcher has some questions, she asked them to be more precise and provide further details to understand the context.
4. At the end of the interview, the researcher thanked the interviewees for their time and efforts and told them that she would send them the interview transcripts to make any comments or add further information if they want.
5. Only one interviewee asked for a transcript and the researcher emailed the interviewee both the Arabic and English transcripts of his interviews, and he was happy with the drafts.

**Appendix (2): Interview Questions**

1. **I know that the holding company has unique projects that might be successful or facing some challenges to survive! Could you please tell me about it?**
2. *Aims and goals.*
   1. What are your main yardsticks, expectations and goals of project success in general?
3. *Financial role and non-financial role.*
4. Do you think that the project was successful? Do you think that it exceeded the decision-makers’ aspirations and goals?
5. What were the factors that have contributed to the success of this project? And what were the main challenges that you have faced with this project?
6. Why did you decide to retain the project?
7. Have you achieved your financial goals?  Have you achieved your non-financial goals?
8. How did the financial factors play a role in retaining the project and investing more on it?
9. Why did you decide to continue although you knew that the project was underperforming (for MASH and JC projects)?
10. **How did you feel about the project?**
11. Are you satisfied with the outcomes of the project?
12. What was your role in the retention decision of the project?
13. What were your feelings towards the project every time you achieved a goal?
14. Have you ever felt that you intend to stick with this project no matter what problems are encountered?
