# Supplementary material for: The associations of anger and hope with project retention decisions: A case study
Source: PLoS One. 2023 Apr 19;18(4):e0283322. doi: 10.1371/journal.pone.0283322 (PMC10115263; doi:10.1371/journal.pone.0283322)
Supplement: S2 File — (DOCX) [file pone.0283322.s002.docx]

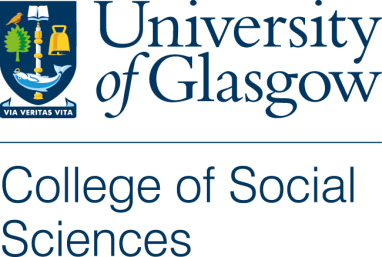
Supporting Information 2

# Participant Information Sheet

# The Influence of Anger and Sunk Cost Bias on Project Status: An Extension to Portfolios of Projects

by Heba Balatia

a PhD researcher in Management

You are being invited to take part in a research study. Before you decide, it is important for you to understand why the research is being done and what it will involve. Please take time to read the following information carefully and discuss it with others if you wish. Ask us if there is anything that is not clear or if you would like more information. Take time to decide whether or not you wish to take part.

Thank you for reading this.

This research aims to study the relationship between anger as one specific emotion and the retention/termination decisions. It will also, highlight the influence of sunk cost bias and portfolio considerations on the anger – retention/ termination decision relationship.

Interviewees will be conducted with top and middle management where each interview will not exceed 60 minutes.

This research will focus on the strategies of your company in retaining and terminating projects. It will present your company as one of these companies that survive in emergent markets as a possible example for companies that operate in similar environments.

Please notice that interviewees have the right to refuse participation in the interviews at any time without providing any reasons.

Please consider that participation is voluntary and interviewees’ personal details will be kept confidential. Also, interviewees will be identified by name but will be anonymised at their request. Confidentiality will be respected regarding legal constraints and professional guidelines.

The collected data will be used only for research uses and will be stored in a safe place as well as being destroyed after research is finished, presented as a PhD thesis and published in journals.

The results of this research could be provided through a written summary to all participants or as a copy of the final manuscript if requested.

Please, consider that this project has been considered and approved by the College Research Ethics Committee.

**For further information please contact Ms. Heba Balatia through**[**2294185h@student.gla.ac.uk**](mailto:2294185h@student.gla.ac.uk) **or her supervisors Prof. Trevor Buck and Dr. Joanna Wincenciak by email. Prof. Buck’s email:** [**trevor.buck@glasgow.ac.uk**](mailto:trevor.buck@glasgow.ac.uk) **and Dr. Wincenciak’s email:** [**Joanna.Wincenciak@glasgow.ac.uk**](mailto:Joanna.Wincenciak@glasgow.ac.uk)**. Also, to pursue any complaint please contact the College of Social Sciences Ethics Officer, Dr Muir Houston, through** [**Muir.Houston@glasgow.ac.uk**](mailto:Muir.Houston@glasgow.ac.uk).**Also, to pursue any complaint please contact the College of Social Sciences Ethics Officer, Dr Muir Houston, through** [**Muir.Houston@glasgow.ac.uk**](mailto:Muir.Houston@glasgow.ac.uk).

_____________End of Participant Information Sheet_____________
